# Gene expression analysis of diabetic foot ulcers reveals the potential impact of Levofloxacin on wound healing

**DOI:** 10.1038/s41598-025-33932-5

**Published:** 2025-12-30

**Authors:** Nazia Hassan, Amber Ilyas, Muhammad Farooq Memon, Yasir Shafiq, Syeda Iffat Zahra, Afsheen Arif, Syeda Nuzhat Nawab

**Affiliations:** 1 The Karachi Institute of Biotechnology & Genetic Engineering (KIBGE), University of Karachi, Karachi, Pakistan; 2 The National Center of Proteomics, University of Karachi, Karachi, Pakistan; 3 The Diabetic Wound and Foot Care Center, Civil Hospital, Karachi, Pakistan

**Keywords:** DFU, Nonhealing wound, Foot ulcer, TGFβ-1, NFE2L2, Oxidative stress, Biochemistry, Biotechnology, Genetics, Molecular biology, Endocrinology

## Abstract

Chronic nonhealing wounds in diabetic patients represent a significant and persistent clinical challenge, contributing to the increased morbidity and mortality of patients with diabetes worldwide. The selection of an appropriate treatment strategy is crucial for mitigating these challenges and enhancing clinical outcomes. While antibiotics are commonly employed to manage infections associated with diabetic foot ulcers (DFUs), their over prescription and prolong use remains a contentious issue. There is evidence linking antibiotics to the induction of oxidative stress, which aggravates the already disrupted redox balance in diabetic patients, thereby worsening their cellular functions. However, the molecular mechanisms and effects of these antibiotics on the expression profiles of genes have not been studied before. Therefore, the present study aimed to analyze alterations in the expression of key wound healing-related genes, such as *TGFΒ-1, NFE2L2 (*also called Nrf2*), HMOX1,* and *MMP-9,* following Levofloxacin use in patients with DFUs. Biopsy samples were collected from a cohort of 30 participants and categorized into three groups: DFUs treated with antibiotics (DFU + Ab), DFUs without antibiotics (DFU-Ab), and a control group (C). The gene expression levels were evaluated via reverse transcriptase quantitative PCR (RT‒qPCR). Our findings revealed a significant (p < 0.05) increase in the expression of *TGFβ-1, HMOX-1, NFE2L2*, and *MMP-9* in the presence of antibiotic (Levofloxacin). The increase in the expression of these genes suggests that antibiotics may induce oxidative stress, leading to biological changes through altering the expression of genes involved in oxidative stress and the inflammatory pathway, thus impacting the wound healing process.

## Article Highlights

- Chronic nonhealing woundleading to Diabetic Foot Ulcers (DFUs) represent a significant clinical challenge in diabetic patients.
- While antibiotic therapy is commonly administered to manage infections and facilitate wound healing, its role in inducing oxidative stress remains a subject of ongoing debate.
- Antibiotic-induced disruption of redox homeostasis may impair cellular functions by modulating oxidative stress and inflammatory pathways, thereby influencing the wound healing process.

## Introduction

Diabetic foot ulcers (DFUs) are among the severe complications of diabetes and are characterized by lesions in the deep tissues of the lower limbs. The key risk factors for the development of DFU include high plantar pressure, peripheral neuropathy, peripheral vascular disease, and trauma. Alarmingly, it is estimated that a lower limb or part of it is amputated due to diabetes-related complications every 30 s worldwide, imposing a substantial physical, social, and economic burden^1^. Persistent inflammation and non-healing wounds are the hallmarks of DFUs and pose significant challenge in wound management^2^. Approximately 537 million people have diabetes worldwide, among which 15–20% develop foot ulcers, and 80% of these patients experience diabetic foot infection along with ulceration^3^. Non healing DFU poses a significant socioeconomic burden, with an estimated cost of $4 billion every year. In Pakistan, the prevalence of diabetes is notably high, with 12.16% of diabetic patients affected by DFUs^4^. These statistics highlight the substantial public health challenge that DFUs and impaired diabetic wounds present, especially in regions with a high prevalence of diabetes.

Normal wound healing process is comprised of four overlapping phases including homeostasis, inflammation, proliferation and remodeling. In acute wound, these phases occur in timely manner to ensure closure of the wound at the remodeling stage starting from clotting to stop bleeding; however, in chronic wounds such as DFU, the healing process stalls at one or more phases^5^. Unlike nondiabetic wounds, the diabetic wound has longer inflammatory phase that delays the wound healing due to production of excessive proinflammatory cytokines by macrophages^6^. Moreover, the inflammatory macrophage does not transit to anti-inflammatory macrophage in diabetic wounds^7^. The inflammatory phase is also disrupted by neutrophil whicht releases cytotoxic enzymes, free radicals and inflammatory mediators that further increase the reactive oxygen species (ROS), a condition called oxidative stress. This oxidative stress cause damage to the tissues and delays the healing process^8^. Prolonged inflammation is considered one of the main cause of impaired wound healing in the diabetics. During the early phases in diabetic wound, NETosis (a process in which neutrophil release granular molecular to fight exotic pathogen), is dysregulated resulting in proinflammatory cascade and production of excessive cytokines that delays the wound healing^9^. Moreover, hyperglycemia results in production of Advanced Glycated End products (AGEs) that alter the structure and function of proteins by binding to receptor of advanced glycated end products (RAGE). This activates the nuclear factor kappa-B (NF-Kb) thus prolonging the inflammation process^10^. Along with inflammation, significant changes occur in extracellular matrix (ECM) that perpetuates the non-healing DFU. The aberration in collagen metabolism such as dysregulation of collagen degrading enzymes called the matrix metalloproteinase (MMPs) are excessively expressed, resulting in proteolytic environment that lowers the collagen content, making ECM inefficient to support wound closure^11^. Similarly, the process of angiognesis is also disrupted in diabetic wound. The process of angiognesis that is necessary for both formation of granulation tissue and providing oxygen and nutrition, is disrupted because the angiogenic growth factors are reduced^12^. This reduced growth factors are possibly due to reduced macrophages^12^. The remodeling phase also differs in diabetic wound because the scars are characterized by lower collagen synthesis and alteration in structure compared to non-diabetic wounds^13^. The remodeling phase is also altered in diabetics due to reduced collagen synthesis, impaired fibroblast activity and change in collagen structure thus resulting in poor wound contraction, weak scar tissue and low tensile strength, ultimately disrupting the normal scar formation^9^.

Reactive oxygen species (ROS) plays a pleiotropic role in the various phases of the wound healing process by implicating in multiple pathophysiological functions such as fighting microbes as well as acting as secondary messenger to regulate angiongesis, cell growth, and Extracellular matrix (ECM) disposition^14^. During the haemostasis phase, Nitrogen oxide (NO) prevents adhesion of platelets to the walls of blood vessels, and ROS such as hydrogen peroxide prompts recruitment of neutrophils and monocytes. In the inflammation phase, ROS prompts activation of immune cells and play vital role in eliminating pathogens to avoid infection. During the proliferatory phase, the ROS modulate cellular signaling pathways to promote proliferation, migration and differentiation of cells like fibroblast and keratinocytes and ultimately promote remodeling of collagen and extracellular matrix (ECM) formation^15^. Considering their role in various cellular processes during the wound healing, it is crucial to tightly regulate the ROS production because too high and too low level of ROS results in abberated wound healing process through tissue damage, pathophysiological stalling, and impeding cellular and molecular process in the wound healing ultimately causing chronicity^16^.

In diabetes, wound healing is affected by various physiological processes, including oxidative stress. Excessive oxidative stress and redox imbalance are the major causes of healing issues in diabetic wounds^17^. Clinically, nonhealing diabetic wounds are characterized by a highly oxidizing environment linked with hyperglycemia and tissue hypoxia^18^. Although the exact role of reactive oxygen species (ROS) in wound healing is not fully understood, major evidence in the literature suggests that ROS are crucial for wound healing not only as a germicide but also as a cellular signal ^2,19^. The balance between the body’s ROS production and that of antioxidant enzymes is crucial for normal wound healing; however, in DFU, the imbalance between the two delays the normal wound healing process. In the clinical management of DFU, multiple strategies are used, including antibiotic therapy. However, long-term use of antibiotics causes drug resistance, which further delays the wound healing process^20^. Therefore, research on the molecular mechanism of the effects of these antibiotics is crucial for improving the clinical outcomes and prognosis of patients with DFU.

A significant factor affecting ROS production is the presence of antibiotics^20^. The increased oxidative stress deleteriously affects the metabolism, structure and blood supply to peripheral nerves in diabetes, resulting in extensive damage to the normal wound healing mechanism^21^. Antibiotics are commonly given to patients with DFU, although their role in this setting is equivocal^22^. In addition to their antimicrobial and bactericidal effects, antibiotics such as quinolones and fluoroquinolones are known to have immunodulatory effects on the production of ROS^22^. A prevalent issue is the over prescription of antibiotics in the absence of clear evidence of infection, with the belief that such treatment will mitigate infection risk and support wound healing^23^ To date, studies have focused on the use of antibiotics for their antimicrobial effects on diabetic foot ulcers. This study aimed to investigate the possible role of antibiotics in modulating the gene expression of key genes, such as TGFΒ-1, NFE2L2, HMOX-1 and MMP-9, during wound healing in patients with DFU.

## Materials and methods

### Clinical specimens

This study was conducted from February 2024 to August 2024 following ethical approval from the Bioethics Committee of the Karachi Institute of Biotechnology and Genetic Engineering (Reference No. KIBGE/ICE/019/29/01/2024). A total of 30 participants, aged 30–65 years, were included in the study. The cohort comprised three groups: 10 diabetic patients with chronic foot ulcers who were receiving Levofloxacin as an antibiotic treatment (DFU + Ab), 10 diabetic patients with chronic foot ulcers without antibiotic treatment (DFU-Ab), and 10 nondiabetic healthy volunteers serving as controls (C). The participants under the antibiotic treated group were using Tablet Levofloxacin (500 mg every 12 h) daily and each patient had been on using this antibiotic for more than three weeks. Biopsy samples were collected from the Civil Hospital Karachi Diabetic Foot Clinic. Informed consent was secured from all participants before sample collection, facilitated by a structured questionnaire. All the procedures involved followed the guidelines of Helsinki. The collected tissue samples were frozen in liquid nitrogen immediately and stored at − 80 °C for further analyses. The demographic characteristics of the study participants are detailed in Table 2.

### RNA extraction and cDNA synthesis

Prior to initiating the experimental procedures, all the working surfaces and materials were thoroughly cleaned with 70% ethanol to ensure a sterile environment. A total of 100 mg of tissue was used for RNA extraction from both the control and diseased groups by using TRIzol Reagent (Invitrogen, Life Technologies, USA) in accordance with the manufacturer’s instructions. The purity and yield of the total RNA were evaluated using spectrophotometer (PerkinElmer, Waltham, MA, USA). The optical density (OD) was measured at 260 and 280nm, and the ratio was measured to determine the purity of the extracted RNA (a ratio > 1.5 was considered indicative of acceptable RNA purity). Following RNA extraction, cDNA synthesis was performed via the reverse transcription of 500 ng of RNA according to the guidelines of the RevertAid First-Strand cDNA synthesis kit (Thermo Fisher Scientific, Massachusetts, USA).

### Relative gene expression by quantitative real-time PCR (RT‒qPCR)

To quantify the mRNA levels in all sample groups, DFU + Ab, DFU-Ab, and corresponding normal tissues (control), a quantitative real-time 7300 PCR system (Applied Biosystem Corp., California, USA) was used. The q‒PCR mixture was composed of SYBR Green master mix, cDNA, nuclease-free water and a pair of gene-specific primers. The real-time qPCR program was as follows: 95 °C for 14 s (denaturation), 60 °C for 1 min (annealing) and 72 °C for 45 s (extension) for 40 cycles. The relative expression of the genes and the control group were studied via a housekeeping gene (glyceraldehyde 3-phosphate dehydrogenase (GAPDH)). The mRNA levels of the selected genes were quantified via the ΔΔCt method^24^. The sequences of the primers are given in Table 1.

**Table 1:** Sequences of the primers used in the RT‒qPCR experiment.

GENES	Forward primer 5′ to 3′	Reverse primer 5′ to 3′
TGF-B1	GTGGAAACCCACAACGAAAT	CGGAGCTCTGATGTGTTGAA
NFE2L2	AAACCACCCTGAAAGCACAG	AGTGTTCTGGTGATGCCACA
GAPDH	ACAGTCAGCCGCATCTTCTT	ACGACCAAATCCGTTGACTC
HMOX-1	TCTCTTGGCTGGCTTCCTTA	TAGGCTCCTTCCTCCTTTCC
MMP-9	AAGCTGGACTCGGTCTTTGA	CCTGTGTACACCCACACCTG

### Functional and pathway enrichment analysis

Gene Ontology (GO) was used to annotate genes as potential biological phenomena, including molecular functions, biological processes and cellular components. GO and KEGG^25^ analyses were performed via the clusterProfiler R package.

### Integration of the protein‒protein interaction (PPI) network

STRING (Search Tool for the Retrieval of Interacting Genes/Proteins)^26^ was used to perform gene interaction analysis to evaluate the functional and physical associations of genes or proteins. The interaction network for Nrf-2, TGFβ-1, HMOX1 and MMP-9 was retrieved from STRING via default settings.

### Statistical analysis

Categorical data are expressed as numerical values, and differences within and between the study groups were analyzed via one-way ANOVA. Variables with a normal distribution are expressed as the mean ± standard deviation (SD), and pairwise comparisons were evaluated via Tukey’s post hoc test. Statistical analyses were conducted via SPSS software (version 20), with the significance level set at *p* < 0.05.

## Results

### Baseline characteristics of the study participants

The study involved 30 participants, consisting of 21 males (70%) and 9 females (30%), stratified into three groups: DFU + Ab (Participants with diabetic foot ulcer using antibiotics), DFU-Ab (participants with diabetic foot ulcer without antibiotics) and control. Table 2 shows the clinical and baseline characteristics of the participants. 70% of the participants were male thus suggesting that diabetic foot ulcer was more prevalent in male compared to female.

**Table 2:** Baseline characteristics of the study participants.

Parameters		Control (*n* = 10)	DFU + Ab (*n* = 10)	DFU-Ab (*n* = 10)	*p* value
Age (Years)		56.1 ± 5.8	58.4 ± 6.2	57.3 ± 6.0	0.62
BMI (kg m^2 −1^)		26.1 ± 2.3	27.0 ± 2.5	28.7 ± 2.1	0.55
FBS (mg/dl)		90.6 ± 2.22	180 ± 12.91	179 ± 11.9	< 0.001***
RBS (mg/dl)		111.4 ± 2.22	286 ± 47.4	275 ± 35	< 0.001***
HbA1c (%)		5.20 ± 0.44	10.9 ± 1.16	9.9 ± 1.37	< 0.001***
Sex (M/F)		4 / 6	8 / 2	7 / 3	–
Duration of diabetes (years)		–	13.2 ± 5.4	12.5 ± 5.0	–
Duration of DFU (months) N(%)	≤ 2	–	4(40)	5(50)	–
	≥ 2	–	6(60)	5(50)	–
Wound size (cm^2^) N(%)	≤ 5	–	3(30)	6(60)	–
	≥ 5	–	7(70)	4(40)	
Wagner grade (2/3/4/5)			1 / 3/4/2	2/5/3/0	–
Ischemia Severity(Mild / Moderate / Severe)			2/4/4	2/5/3	
Antibiotic Treatment (yes/no)			10/0 levofloxacin (500 mg) 2 times daily	0/10	

(Mean ± SD), (*P* value < 0.001 ***).

### Gene expression analysis

**Fig. 1:**
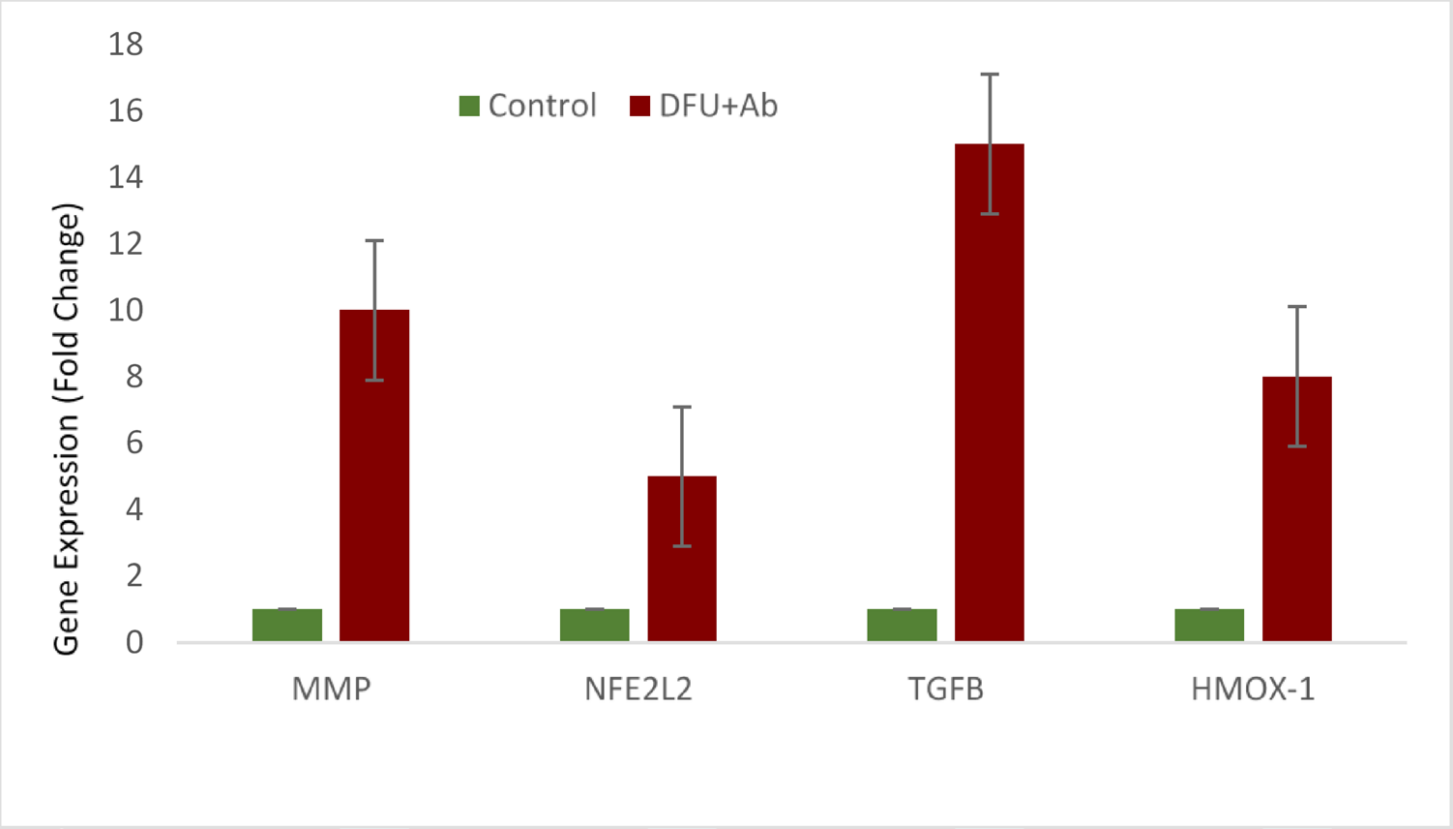
Comparison of relative gene expression levels between Control and DFU+Ab Groups

Figure 1 shows the expression analysis of the target genes (*MMP-9, NFE2L2, TGFβ-1* and *HMOX-1*) in the control and DFU + Ab groups. The fold change represents the relative gene expression level. A significant increase (*p* < 0.05) in gene expression was observed in the antibiotic-treated group (DFU + Ab) compared with the control group, suggesting a potential impact of Levofloxacin on the expression of oxidative stress-related genes (*NFE2L2* and *HMOX-1*) and genes involved in wound healing (*TGFβ-1* and *MMP-9*).

**Fig. 2:**
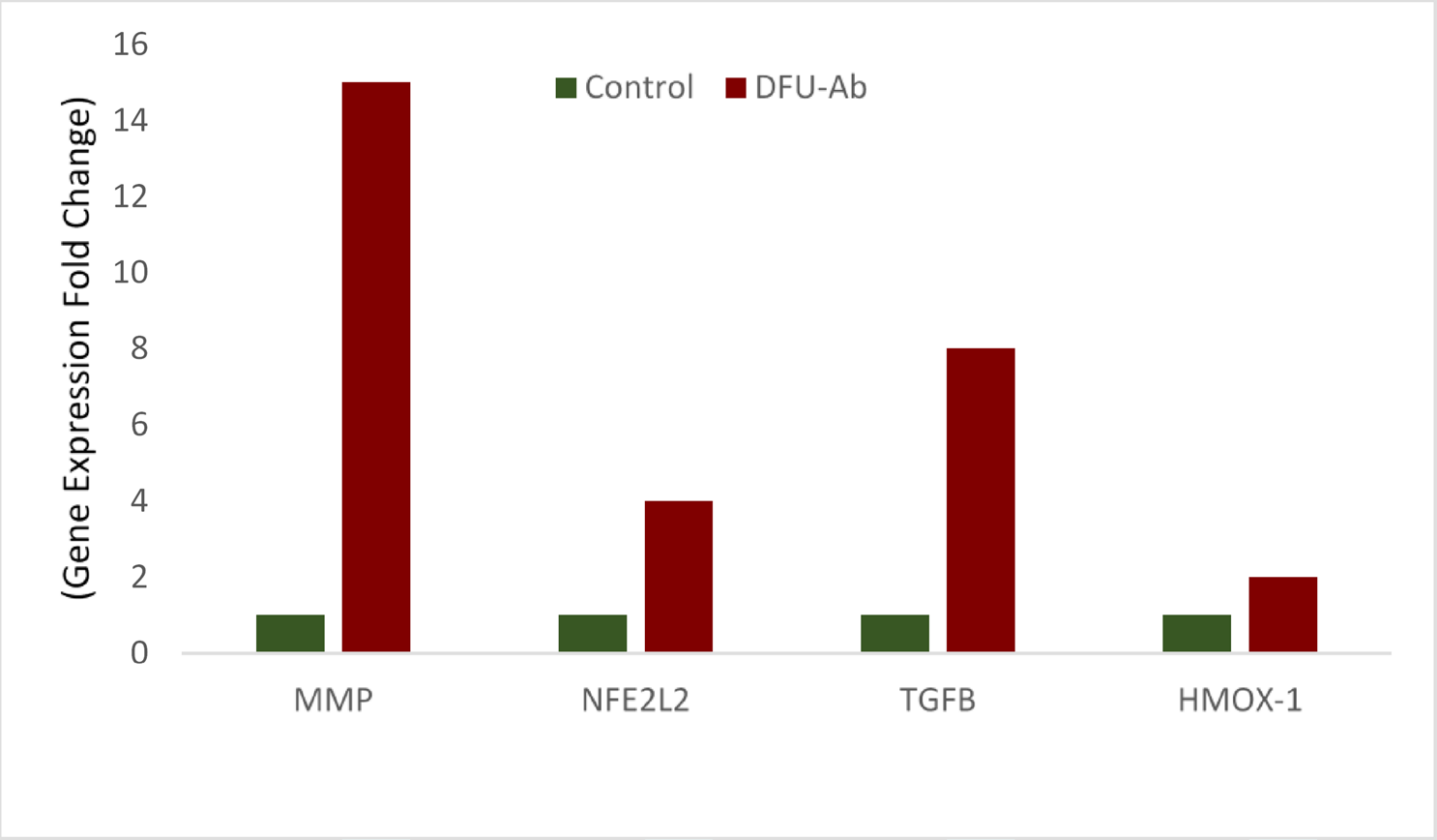
Relative gene expression levels in Control and DFU-Ab Groups

The relative gene expression levels were then compared between the control and DFU-Ab groups, as shown in Fig. 2. The findings showed that in the absence of antibiotic, the expression of these genes was significantly greater (*p* < 0.05*) in DFU patients than in controls. However, the change was less intense than that in the DFU + Ab group, as shown in Fig. 2. These findings highlight the potential impact of antibiotics on the regulation of genes involved in oxidative stress and wound healing pathways in diabetic foot ulcers.

**Fig. 3:**
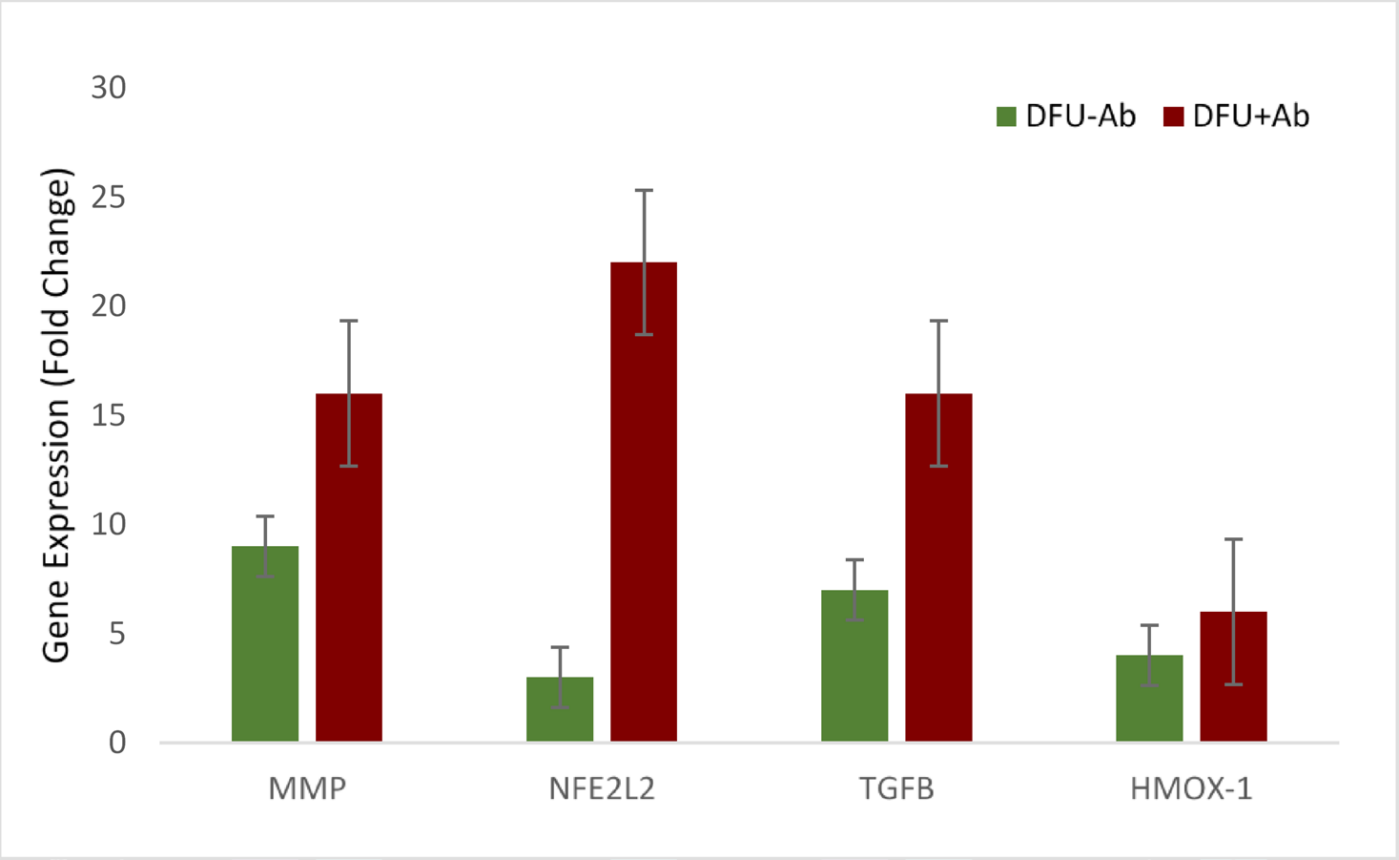
Relative Gene Expression levels in DFU-Ab and DFU+Ab Groups

The relative gene expression level was also evaluated among the two disease groups, DFU-Ab and DFU + Ab. The findings revealed that patients with diabetic foot ulcers who Levofloxacin (DFU + Ab) had significantly greater expression of the target genes (*p* < 0.05*) than did those without antibiotic (DFU-Ab), as shown in Fig. 3.

**Fig. 4:**
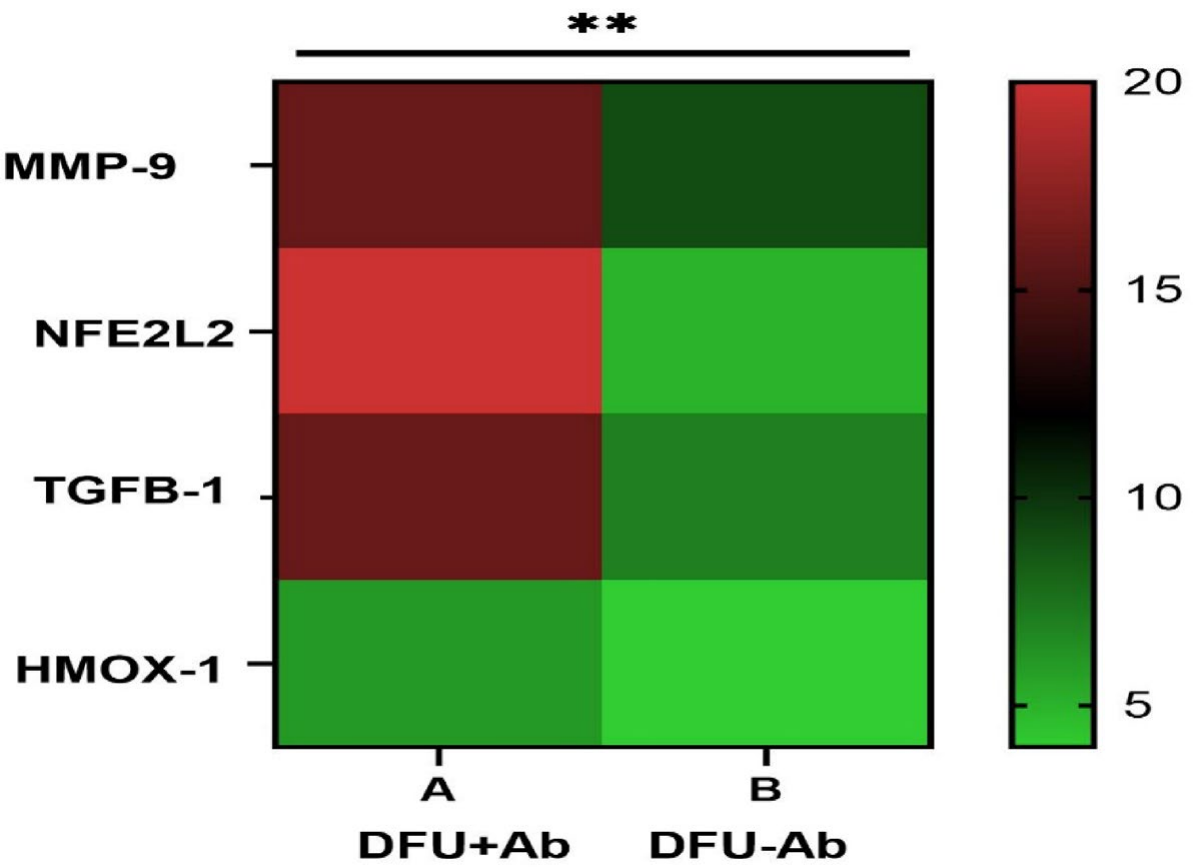
Heatmap of differential gene expression in DFU patients with and without Levofloxacin treatment

A heatmap was also generated to show the relative gene expression via GraphPad Prism. The color gradient shows the expression level of each gene during the wound healing process. The heatmap revealed significant variations in the expression patterns, suggesting that the presence of antibiotics in diabetic foot ulcer patients significantly impacts the expression of genes involved in the response to oxidative stress and the wound healing process (Fig. 4) Heatmap of the expression levels of all four genes (*MMP-9, TGFB1, NFE2L2 and HMOX1*) among the groups (Antibiotic treated (DFU+Ab) and non-antibiotic users (DFU-Ab) are shown where low expression (green) and high expression (red) is shown in Fig 4. The expression of NFE2L2 and TGFB was significantly higher (p<0.05).

### GO and KEGG enrichment analysis

**Fig. 5:**
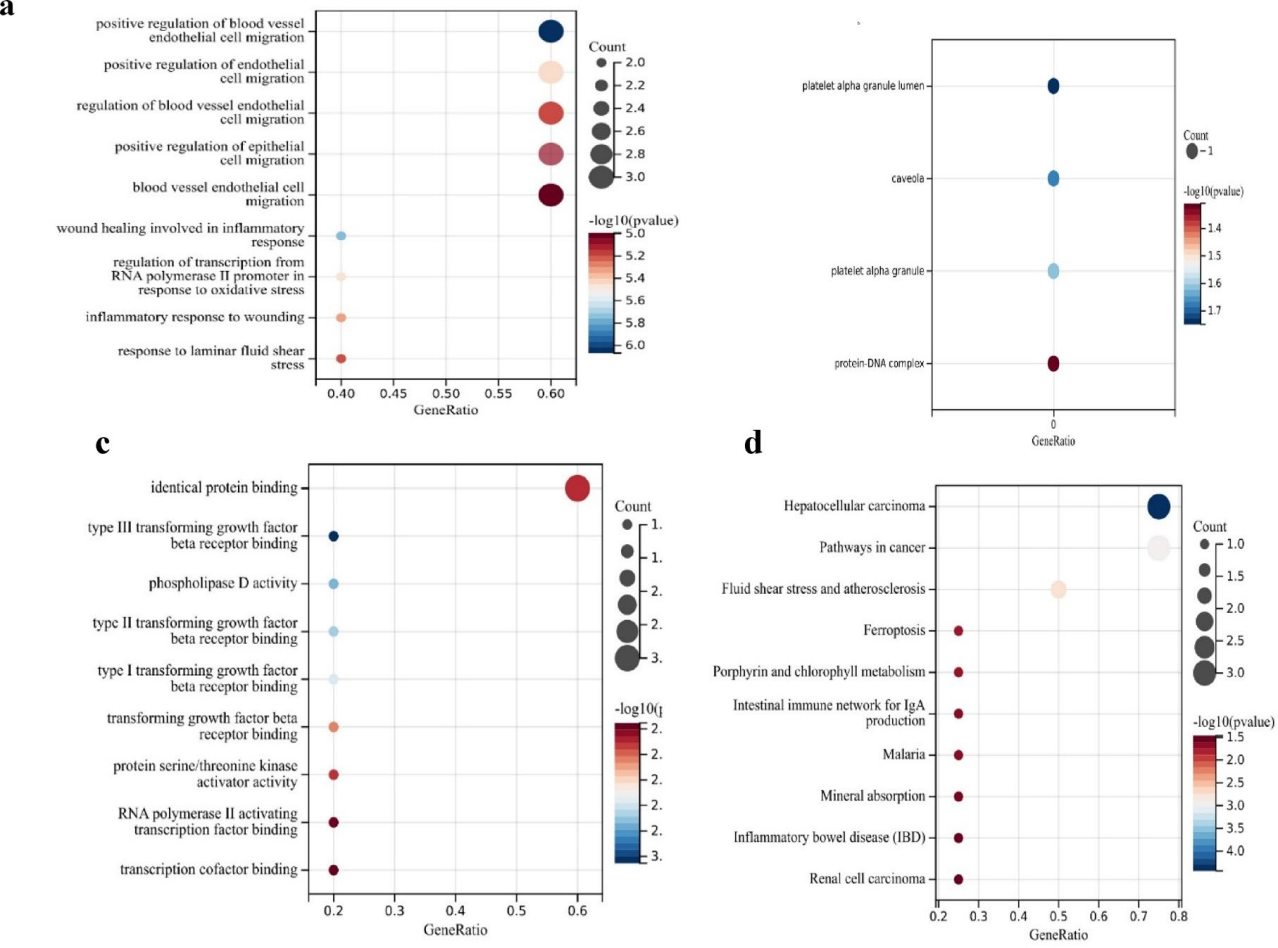
GO and KEGG enrichment analyses are shown in a bubble plot.

Gene Ontology analysis revealed associations of *NFE2L2, HMOX-1, TGFΒ-1* and *MMP-9* with biological processes, cellular components and molecular functions. Three GO category results are presented in Fig. 5a–c. Figure 5a shows the biological process (BP) analysis results depicting the associations of the target genes with cell migration, endothelial function and the regulation of blood vessels. GO biological process analysis revealed that the target genes were enriched in response to stress, the inflammatory response to wounding, the response to oxidative stress and inflammation. Figure 5b shows the localization of the CCs, which were predominantly found in the protein DNA complex, platelet alpha granule lumen and caveolae. GO molecular function revealed that the targets were enriched in identical protein binding, growth factor beta receptor binding, protein/serine/threonine kinase activity and transcription cofactor binding (Fig. 5c). KEGG pathway analysis (Fig. 5d) revealed that the genes are associated with multiple pathways.

### Integration of the protein‒protein interaction (PPI) network

**Fig. 6:**
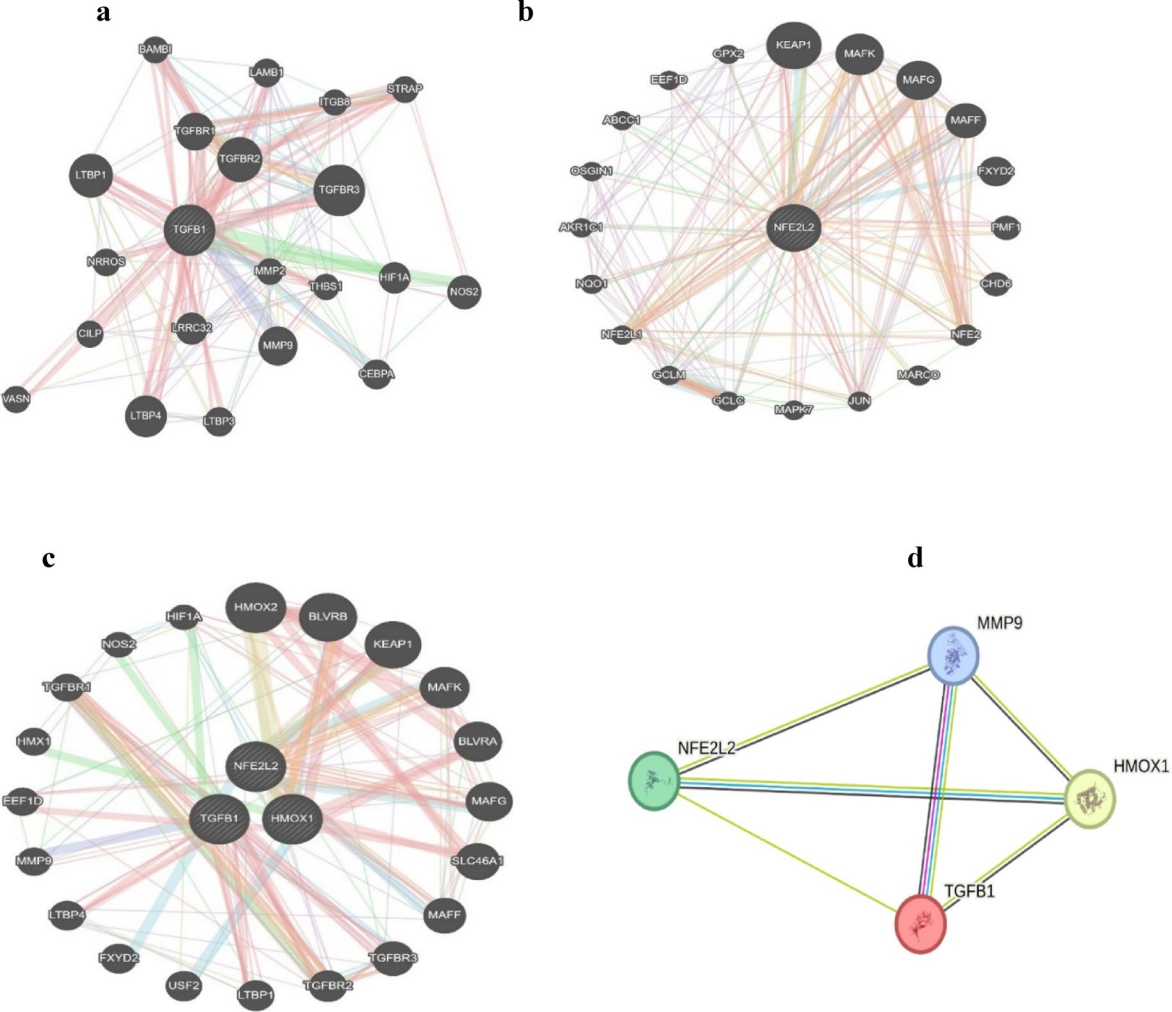
Protein‒protein interaction (PPI) network (**a**): PPIs of TGFΒ-1, (**b**) PPIs of NEF2L2, (**c**) PPIs among HMOX-1, TGFΒ-1 and NFE2L2 and (**d**) PPIs among MMP-9, TGFΒ-1, HMOX-1 and NFE2L2.

The interaction network for Nrf-2, TGF-beta, HMOX1 and MMP-9 was retrieved from STRING via the default settings (Fig. 6). The networks of gene interactions were visualized via the MANIA gene. The figure shows that the target genes are functionally associated with each other.

## Discussion

DFUs tend to heal poorly, resulting in delayed or non-healing scars that require intensive and prolonged treatment, resulting in a high risk of amputation and even death. The present study revealed that the percentage of male participants with DFU was greater than that of female participants. This can be attributed to sex differences in self-care and disease awareness, inappropriate footwear, and delays in seeking professional and medical care.This finding aligns with a previous study showing that DFU is more prevalent among men than among women with diabetes^27^. The study revealed that age and Body mass index (BMI) were not significantly different among the study group indicating that the participants had similar anthropometric and demographic characteristics. The finding also suggests that the change in molecular outcomes are unlikely to be impacted by baseline characteristics in BMI or age^28^. The findings revealed that patients with DFU had longer duration of diabetes. This may be attributed to persistent hyperglycemia and the prevalence of peripheral neuropathy and arterial diseases, which are key contributors to the development of DFU, in aged patients^29,30^. Considering the clinical parameters, the present study revealed that HbA1c, FBS and RBS are significantly associated with DFU compared with controls. These findings are consistent with earlier reports suggesting that higher HbA1c and FBS are strongly linked with the severity and complications of diabetes, including DFU^31,32^.

Antibiotic therapy represents a critical component of the multifaceted management strategies employed for DFUs. Multiple bactericidal antibiotics are known to provoke ROS production via mitochondrial dysfunction in mammalian cells^33–35^. However, limited data exists on the role of antibiotics in DFU. To the best of our knowledge, this study is the first to evaluate gene expression in patients with diabetic foot ulcers receiving antibiotic treatment. This study specifically evaluated the impact of antibiotic (Levofloxacin) therapy on the expression of genes involved in oxidative stress and wound healing. The present study revealed a significant (p < 0.05*) increase in the expression of *NFE2L2, TGFβ-1, HMOX-1*, and *MMP-9* in the antibiotic-treated groups compared with the control groups (Fig. 1), suggesting that antibiotics may induce oxidative stress that alters gene expression at the wound site in diabetic foot ulcers.

NFE2L2 (Nrf2) is a key transcription factor that regulates the antioxidant response and angiogenesis. Its upregulation occurs alongside TGFβ-1 during oxidative stress to reduce damage and suppress inflammation in diabetic wounds^36^. Pathologic oxidative stress not only harms the phases of wound healing (inflammation, proliferation, and remodeling), making these unable to proceed seamlessly but also leads to worsening of the wound site through infection and neuropathy in DFUs^37^. Considering the induction of oxidative stress by antibiotics, the findings of the present study revealed a significant increase in the expression of NFE2L2 at the wound site in the antibiotic-treated group. This suggests that although NFE2L2 has role in anti-inflammatory and antioxidant response, the increase in its expression contributes to chronic inflammation thus, impacting the wound healing process. NFE2L2 regulates the antioxidant response to restore redox balance; however, excessive or persistent activation in the context of diabetes might impact the wound healing process in DFUs. The significant increase in NFE2L2 and HMOX-1 suggested an increase in oxidative stress, possibly caused by antibiotics, which was greater in the DFU + Ab group (p < 0.05). This finding is supported by a study showing that antibiotics cause oxidative stress and damage the mitochondrial membrane in various mammalian cells, leading to deleterious effects on normal functioning and physiology ^38^. The Nrf2/Keap1 pathway is typically activated in response to oxidative stress in a dose-dependent manner. However, under hyperglycemic conditions and excessive oxidative stress, the capacity of Nrf2 to bind with the Maf protein in the nucleus is reduced, leading to impaired Nrf2 function^39^. The impairment of Nrf2 activation exacerbates ROS production, causes abnormal inflammation at the wound surface and stagnates the wound healing process during the inflammatory phase^38,39^. Impaired *NFE2L2* activation in diabetic wounds prolongs inflammation and reduces the antioxidant response, which is exacerbated by antibiotic-induced ROS^40,41^. This finding suggests that oxidative stress impairs DFU healing, complicating recovery of diabetic wounds^42^. Hemeoxygenase-1 (HMOX-1), which is an antioxidant factor regulated by Nrf2, plays a crucial role in preventing oxidative stress. It is significantly more common in patients with diabetes and diabetic foot ulcers^43^. The results of the present study revealed greater expression of HMOX-1 in patients with DFU than in controls (Figs. 1 and 2). Compared with those in the DFU + Ab and DFU-Ab groups, HMOX-1 expression was high in patients receiving Levofloxacin (Fig. 3), highlighting the possible role of antibiotics in inducing oxidative stress, which results in an increased antioxidant response.

TGF-β, a profibrotic cytokine, promotes the deposition of extracellular matrix and tissue remodeling along with its crucial role in cell differentiation, migration, apoptosis and proliferation; however, excessive activation could impair the normal wound healing process^44^. The findings of this study revealed a significant increase in TGF-β expression in the antibiotic-treated group (Fig. 3). Antibiotics, while critical for infection control, appear to intensify oxidative stress, as observed by increased ROS markers and the upregulation of antioxidant-related genes^45^. The findings of this study are also consistent with those of a previous study showing dysregulation of the TGF-beta pathway in diabetes and its complications^46^. TGF-β is a crucial regulator of wound healing and is released by platelets in the early stages of the wound healing process. These findings are consistent with a previous study showing that TGF-β was significantly greater in patients with DFU than in controls^47^. Under hyperglycemic conditions, where impaired redox balance is prevalent, an increase in TGF-β expression is observed. Our findings demonstrated that the levels of TGF-β were markedly greater in patients with diabetic foot ulcers (DFUs) than in the control group (Figs. 1 and 2). Furthermore, a comparative analysis between the DFU + Ab (group using Levofloxacin)) and DFU-Ab (without antibiotics) groups revealed notable upregulation of TGF-β in the DFU + Ab group (Fig. 3). These findings underscore the possible potential of antibiotics in increasing oxidative stress in these patients. Similarly, the findings revealed that the MMP-9 level was also significantly greater in the group treated with antibiotics (Fig. 3). The increase in the expression of these genes suggests that antibiotics may induce biological changes through altering the expression levels of genes involved in oxidative stress and the inflammatory pathway, thus impacting the wound healing process. The increase in MMP-9, however, is not consistent with a previous study showing that an increase in NFE2L2 significantly increased TGFB1 and reduced MMP-9 during diabetic wound healing^48^.

**Fig. 7:**
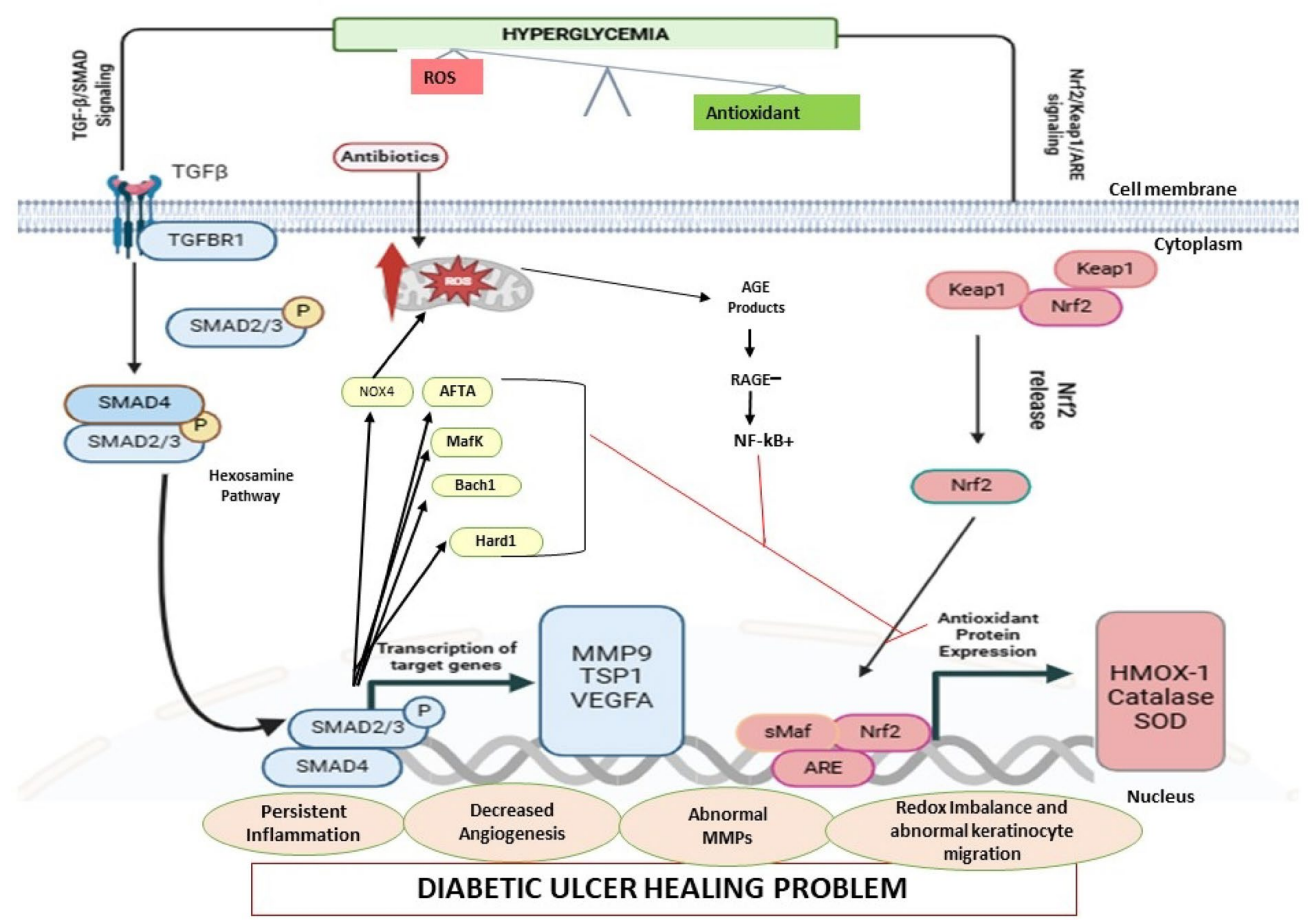
Complex cross-talk between TGF-β/SMAD signaling (shown in blue) and the Nrf2/Keap1 signaling pathway (shown in red) is activated as a result of hyperglycemia and oxidative stress. The already imbalanced redox state in diabetic wound might be further exacerbated by antibiotic-induced oxidative stress. The TGF-β pathway leads to the induction of NADPH oxidase 4 (Nox4), which results in the production of more ROS and increases the intensity and duration of TGF-β signaling. An increase in TGF-β signaling increases AFT3, Bach1 and MafK, which are reported to downregulate the activity of Nrf2. The literature also shows that increasing the activity of the TGF-β signaling pathway via ROS production allows JNK and p38 MAPK (not shown) to lower Nrf2 activity. Increased Nrf2 can also inhibit TGF-β1 function by blocking smad3 activation (not shown). Dysregulation of the expression of these genes results in altered expression and function of various genes, causing persistent inflammation, redox imbalance, and low angiogenesis ultimately causing abberated wound healing process in DFU.

Heatmap analysis (Fig. 4) revealed higher expression of *NFE2L2* and *TGFB1* in response to antibiotic treatment compared to DFU-Ab group, suggesting the potential role of antibiotic in modulating oxidative stress. Both these genes are involved in oxidative stress response and wound healing processes. Bioinformatics analysis also revealed a strong link between *NFE2L2, HMOX-1, TGFβ-1,* and *MMP-9*, highlighting their collective role in the wound healing process  (Figs. 5 and 6). Aberrant expression of growth factors, chemokines, and transcription factors delays healing, with oxidative stress being a major contributor. Growth factors such as *TGFβ-1* recruit inflammatory cells and promote ECM synthesis during early wound healing^49^. Hyperglycemia activates pathways such as the NF-κB pathway, leading to the overexpression of *TGFβ-1* and thrombospondin-2, further delaying the healing process^50^. Antibiotic-induced ROS cause oxidative stress, damaging mammalian and bacterial cells^51^. The proposed mechanism of action of the key genes involved in wound healing and the role of antibiotics in inducing oxidative stress are presented in Fig. 7. The already imbalanced redox state of the diabetic wound might be further impaired by antibiotic-induced oxidative stress because bactericidal antibiotics are known to induce mitochondrial dysfunction and elevate ROS production that could impact the expression of wound healing genes^36^. There could be other factors contributing in increasing the ROS production therefore, it is recommended that the ROS related mechanism is validated through cell-based experiments. The notion that antibiotics effect only bacteria has now altered as studies show that certain antibiotics may also damage mammalian cells thus posing a significant challenge for patients on long-term antibiotic therapy^36^. In human, the oxidative cellular damage and oxidative stress due to bactericidal antibiotics underlies various adverse side effects^52^. The mechanism for this has been shown that bactericidal antibiotics cause mitochondrial disruption by disturbing mitochondrial electron transport chain, that could lead to ROS production. The findings of this study aligns with those of^53^ which revealed that oral antibiotics significantly modulated gene expression in gut and liver. Antibiotic-mediated modulation within the cells comprises of complex processes at biochemical, ultrastructural and biochemical levels after the drug-target interaction^54^. This finding also corroborates previous finding that under hypoxic condition, antibiotic intervention exacerbates oxidative stress as well as the inflammation in rats^55^. Although this study presents only one class of antibiotic, investigation on wide range of antibiotics is recommended before conclusion is drawn about the category and effect of the antibiotics on the diabetic wound healing. We observed a marked difference in the expression of wound healing genes between the antibiotic and non-antibiotic users, the mechanism for this difference needs to be disentangled so full understanding is gained.

## Conclusion

This study revealed that antibiotic treatment may potentially alter the expression of key genes involved in wound healing and antioxidant pathways possibly by inducing ROS production. Oxidative stress plays a critical role in activating the TGF-β/Smad and Nrf2/Keap1 signaling pathways; however, persistent ROS production and the overexpression of these genes may alter the normal function of these genes, resulting in delayed or nonhealing diabetic wounds. The present study shows the role of antibiotics in possible cause of oxidative stress resulting in altered wound healing process, highlighting strategies to dull this damage and improve safe antibiotic treatment therapy. The potential role of antibiotic induced ROS production could further be validate through cell-based experiments as other contributing factors might also play role in the ROS production. Further research is also needed to explore the cross-talk between oxidative stress, *NFE2L2*, and *TGF-β* in delaying the process of wound healing in diabetic foot ulcers. Additionally, combining antioxidants with antibiotics could enhance therapeutic efficacy, warranting further investigation to improve patient outcomes and quality of life.

## Data Availability

Data is provided within the manuscript.
